# Overview of rabies post-exposure prophylaxis access, procurement and distribution in selected countries in Asia and Africa, 2017–2018

**DOI:** 10.1016/j.vaccine.2019.04.024

**Published:** 2019-08-27

**Authors:** N. Sreenivasan, A. Li, M. Shiferaw, C.H. Tran, R. Wallace, J. Blanton, L. Knopf, B. Abela-Ridder, T. Hyde, Working group on Rabies PEP logistics, U.R. Siddiqi, S. Tahmina, K. Penjor, L. Sovann, Y. Doeurn, K. Sim, V. Houssiere, M. Tejiokem, R. Mindekem, L. Yu, Y. Wenwu, J. Benié, M. Tetchi, I. Tiembre, A. Deressa, A. Haile, B. Hurisa, N.A. Yawson, S.A. Ohene, M.K. Sudarshan, A. Narayana, A. Mwatondo, S.M. Thumbi, G. Edosoa, L. Baril, R. Ramiandrasoa, M. Rajeev, M.S. Fofana, A. Traore, M. Matchaya, J.L. Burdon Bailey, G. Yale, A. Dolgorkhand, N. Tsogbadrakh, A. Ochirpurev, K. Shrestha, J. Balami, H. Qureshi, N. Salahuddin, E. Villalon, L. Blumberg, A. Gunesekara, Joel Changalucha, H. Nguyen

**Affiliations:** 1 Centers for Disease Control and Prevention, Atlanta, USA; 2 PHI/CDC Global Health Fellowship and ASPPH/CDC Allen Rosenfield Global Health Fellowship, Atlanta, USA; 3 World Health Organization, Geneva, Switzerland; a Disease Control Unit, Communicable Disease Control, Directorate General of Health Services, Bangladesh; b Dewathang Military Hospital, Department of Medical Services, Ministry of Health, Thimphu, Bhutan; c Communicable Disease Control Department, Ministry of Health, Phnom Penh, Cambodia; d World Health Organization, Cambodia; e Centre Pasteur du Cameroun, Cameroon; f Ministère de la Santé Publique, N’Djaména, Chad; g Chinese Center for Disease Control and Prevention, Beijing, China; h Institut National de l’Hygiène Publique de Côte d’Ivoire, Cote d’Ivoire; i Ethiopian Public Health Institute, Ethiopia; j Ministry of Health, Ghana; k World Health Organization, Ghana; l Association for Prevention and Control of Rabies in India (APCRI), Bangalore, India; m Department of Community Medicine, Kempegowda Institute of Medical Sciences (KIMS), Bangalore, India; n Zoonotic Disease Unit, Ministry of Health, Nairobi, Kenya; o Paul G. Allen School for Global Animal Health, Washington State University/Kenya Medical Research Institute, Nairobi, Kenya; p Ministère de la Santé Publique, Antananarivo, Madagascar; q Institut Pasteur de Madagascar, Antananarivo, Madagascar; r Department of Ecology and Evolutionary Biology, Princeton University, United States; s Direction Nationale de la Santé, Mali; t Laboratoire Central Vétérinaire, Bamako, Mali; u Ministry of Health, Malawi; v Mission Rabies, United Kingdom; w Ministry of Health, Mongolia; x National Center for Zoonotic Diseases, Mongolia; y World Health Organization, Mongolia; z Himalayan College of Agricultural Sciences and Technology, Kathmandu, Nepal; aa Department of Public Health, Federal Ministry of Health, Nigeria; ab Pakistan Health Research Council, Islamabad, Pakistan; ac The Indus Hospital, Karachi, Pakistan; ad National Rabies Prevention and Control Program, Department of Health, Manila, Philippines; ae Centre for Emerging, Zoonotic and Parasitic Diseases, National Institute for Communicable Diseases, Johannesburg, South Africa; af Ministry of Health, Colombo, Sri Lanka; ag Ifakara Health Institute, Dar es Salaam, Tanzania; ah National Institute of Hygiene and Epidemiology, Ministry of Health, Hanoi, Viet Nam

**Keywords:** Human rabies, Rabies post-exposure prophylaxis, Rabies vaccine access

## Abstract

**Background:**

Rabies is a neglected zoonotic disease with a global burden of approximately 59,000 human deaths a year. Once clinical symptoms appear, rabies is almost invariably fatal; however, with timely and appropriate post-exposure prophylaxis (PEP) consisting of wound washing, vaccine, and in some cases rabies immunoglobulin (RIG), the disease is almost entirely preventable. Access to PEP is limited in many countries, and when available, is often very expensive.

**Methods:**

We distributed a standardized assessment tool electronically to a convenience sample of 25 low- and middle-income countries in Asia and Africa to collect information on rabies PEP procurement, forecasting, distribution, monitoring and reporting. Information was collected from national rabies focal points, focal points at the World Health Organization (WHO) country offices, and others involved in procurement, logistics and distribution of PEP. Because RIG was limited in availability or unavailable in many countries, the assessment focused on vaccine. Data were collected between January 2017 and May 2018.

**Results:**

We received responses from key informants in 23 countries: 11 countries in Asia and 12 countries in Africa. In 9 of 23 (39%) countries, rabies vaccine was provided for free in the public sector and was consistently available. In 10 (43%) countries, all or some patients were required to pay for the vaccine in the public sector, with the cost of a single dose ranging from US$ 6.60 to US$ 20/dose. The primary reason for the high cost of the vaccine for patients was a lack of funding at the central level to subsidize vaccine costs. In the remaining 4 (17%) countries, vaccine was provided for free but was often unavailable so patients were required to purchase it instead. The majority of countries used the intramuscular route for vaccine administration and only 5 countries exclusively used the dose-sparing intradermal (ID) route. Half (11/22; 50%) of all countries assessed had a standardized distribution system for PEP, separate from the systems used for routine childhood vaccines, and almost half used separate storage facilities at both central and health facility levels. Approximately half (9/22; 41%) of all countries assessed reported having regular weekly, monthly or quarterly reporting on rabies vaccination.

**Conclusions:**

While all countries in our assessment had rabies vaccines available in the public sector to some extent, barriers to access include the high cost of the vaccine to the government as well as to patients. Countries should be encouraged to use ID administration as this would provide access to rabies vaccine for many more people with the same number of vaccine vials. In addition, standardized monitoring and reporting of vaccine utilization should be encouraged, in order to improve data on PEP needs.

## 1. Introduction

Rabies is a neglected zoonotic disease with the majority of human rabies cases caused by the Rabies lyssavirus. Human rabies results in an acute progressive encephalitis that is almost invariably fatal once clinical symptoms appear. The global burden of human rabies is estimated at 59,000 deaths a year, with the greatest burden occurring primarily in Asia and Africa among children in rural areas [1,2]. Up to 99% of human cases are dog-mediated; one of the main strategies of rabies control is ensuring high vaccination coverage in the dog population [2,3]. However, rabies in humans can also be prevented with timely and appropriate administration of post-exposure prophylaxis (PEP). Without PEP, an estimated three million people worldwide would die every year from rabies [2]. Specific steps recommended for PEP depend on the category of exposure to a suspect rabid animal; steps include immediate and thorough wound washing, followed by vaccination with rabies vaccine, and in some cases, administration of rabies immunoglobulin (RIG) [4,5]. When given appropriately and in a timely manner, rabies PEP is almost 100% effective in preventing the disease [4].

The World Health Organization (WHO) currently only recommends the use of purified cell-culture and embryonated egg-based rabies vaccines (CCEEVs) [4-6]. Nerve-tissue based vaccines are no longer recommended due to the possibility of severe adverse reactions, and lower immunogenicity than CCEEVs [4-6]. CCEEVs can be administered by the intradermal (ID) route or the intramuscular (IM) route; vaccination schedules vary depending on the route of administration [4]. However, ID administration requires a fraction of the IM dose over a shorter period of time [4]. Despite the existence of an effective vaccine, many people needing PEP lack access to it [7]. Common barriers to access include the high cost of the vaccine and RIG, and limited availability particularly outside major urban areas [8]. Rabies PEP were previously assessed for a potential investment by Gavi, the Vaccine Alliance (Gavi), in 2008 and 2013 [9]. However, rabies PEP investments were not approved, in part due to a lack of published information on how rabies PEP is provided and distributed particularly in low-income countries, how rabies vaccine needs are forecast, and what systems are in place to monitor their use. Instead, Gavi chose to invest in a learning agenda to fill in some of these knowledge gaps [9]. As part of the 2013 Gavi learning agenda, we conducted an assessment to better understand the systems used for PEP distribution among countries in Asia and Africa. The objectives of the assessment were to describe provision, delivery and distribution systems for rabies vaccine, and to identify areas that could be strengthened across countries with additional investments.

## 2. Methods

We conducted programmatic assessments consisting of a series of interviews with key informants from various groups within the Ministry of Health (MoH), WHO country offices, and from health facilities that provide PEP, using a descriptive assessment tool in a convenience sample of countries in Asia and Africa. The tool was developed for the purposes of the assessment. Key informants included national rabies prevention and control program managers or rabies focal points in countries without rabies control programs, and logisticians or others responsible for procurement, logistics and distribution of rabies biologicals. The assessment tool was administered at the central level; if possible, we visited health facilities and interviewed health workers responsible for delivering PEP. In some countries, health facilities were not visited specifically for this assessment, but were visited by national rabies focal points as part of their routine work; information collected during these routine visits was included in the assessments. In one country (Ethiopia), data was primarily collected as part of a larger rabies prevention and control program under the Global Health Security Agenda (GHSA) umbrella [10].

The assessment tool was mailed electronically to key informants with follow-up conducted via phone, over email or in person. The assessment tool consisted of questions grouped into categories focusing on program delivery; vaccine procurement, requests and distribution; cold-chain; vaccine storage; and vaccine forecasting, monitoring and utilization. At health facilities, cold chain and vaccine storage practices were assessed, and vaccination registers and stock monitoring tools were reviewed to understand rabies vaccine monitoring and reporting at the local level. The assessments were primarily descriptive but we identified key characteristics from each of the major categories to provide a standardized description of programs across countries.

The assessment tool was distributed to 25 countries from the Eastern Mediterranean (1), South East Asian (6), Western Pacific (5), and African (13) WHO regions. We selected a convenience sample of countries including a combination of low- and middle-income countries; Gavi-eligible, transitioning and non-eligible countries; and countries with both a high and low burden of rabies [2,11]. Data were collected between January 2017 and May 2018. The primary focus of the assessments was to describe rabies vaccine distribution in the public sector and other non-private health facilities (e.g., non-governmental organization (NGO)-supported health facilities, or facilities run by research institutions that support governments).

### 2.1. Definitions

Three categories combining vaccine availability and cost were used to describe access to vaccine and RIG: widely accessible, accessible, and limited accessibility. Widely accessible was defined as vaccine or RIG being available for free or at a subsidized cost at the central level, provincial, state or regional level, and at least one health facility in every district, county or zone (or the equivalent administrative level). Accessible was defined as vaccine or RIG being available at the central level and provincial, state, or regional level but not in every district, county or zone, or available in every district but at a cost to patients (greater than US $5/dose), making it less accessible. Limited accessibility was defined as vaccine or RIG only being available at the central level (regardless of cost) or being sporadically available at lower levels because of budget constraints or stock outs.

Countries in which ID administration was introduced only at select health facilities as part of a time-limited pilot project, but where all other health facilities continue to use IM administration (as per national guidelines), were categorized as using IM administration only. We defined prequalified (PQ) vaccines as vaccines that meet the WHO requirements for quality, safety and efficacy and were on the WHO prequalification list [12,13].

We defined Gavi-eligible countries as countries with a Gross National Income (GNI) per capita below or equal to US$ 1580 on average over the past three years [11]. For the purposes of this assessment, we categorized India and Nigeria as Gavi-eligible as they continue to receive funding from Gavi, and categorized transitioning countries as non-eligible.

## 3. Results

### 3.1. Response rate and national programs

We received responses and agreement to participate from key informants in 23 countries: 11 countries in Asia and 12 countries in Africa, resulting in a response rate of 92%. However, not all questions were answered by all responding countries, resulting in changes in denominators for each indicator. The countries assessed included: Bangladesh, Bhutan, Cambodia, Cameroon, Chad, China, Cote d’Ivoire, Ethiopia, Ghana, India, Kenya, Madagascar, Mali, Malawi (Blantyre district only), Mongolia, Nepal, Nigeria, Pakistan, Philippines, Tanzania, South Africa, Sri Lanka, and Vietnam. Of these, 16 countries were Gavi-eligible (Table 1). Focal points at the central level were interviewed in 22 countries; because only one district in Malawi was assessed, this country was only included in the denominator when responses were applicable to the country as a whole or referred to national policies/guidelines. Health facilities were visited in 16 (70%) of 23 countries, either for the purposes of the assessment or as part of routine visits for rabies control. Of 22 countries, 13 (57%) countries (7 countries in Asia and 6 countries in Africa) reported having a national program, a national strategy or guidelines for rabies control and prevention (Table 1).

### 3.2. Availability and cost of post-exposure prophylaxis

Rabies vaccine was available in all 23 countries in the public sector; however, consistency and affordability of the vaccine varied greatly. In 10 (43%) of 23 countries with available rabies vaccine, it was reported to be consistently provided for free in the public sector (Table 1). In an additional four (17%) countries, vaccine was provided free of cost when available, but these countries reported limited capacity to procure vaccines due to insufficient funds, so patients were typically required to purchase it at a pharmacy or in the private sector. In an additional two countries, certain patient groups (e.g. children, people living below the poverty line etc.) could reportedly access the vaccine for free in select health facilities. In the remaining 7 (30%) countries, patients were required to pay for the vaccine in the public sector, with the cost of a single dose of vaccine ranging from US$ 6.60–$20.00/dose (Table 1). Within a country, the cost per dose could vary, depending on the brand of vaccine used. The primary reason for the high cost of the vaccine to patients, cited by all 13 (57%) countries that did not provide vaccine for free, only provided it for free to certain patient groups, or only provided it for free when available, was a lack of funds at the central level needed to subsidize the cost.

RIG was consistently available in 8 (35%) of 23 countries. An additional 12 (52%) countries reported having RIG on a limited basis with frequent stock-outs, primarily due to its high cost and limited global availability. In these countries, health workers prioritize its use based on location and severity of the bite, age of the patient, type of wound, and status of the animal that inflicted the bite. In 3 (13%) of 23 countries, RIG was not available in the public sector. In 6 (26%) of the 20 countries which sometimes or consistently had RIG, it was provided for free when available. In the remaining countries where patients were required to pay the full cost of RIG, the cost per vial ranged from US$ 5 to $70 (Table 1).

### 3.3. Access to PEP

**Fig. 1.**
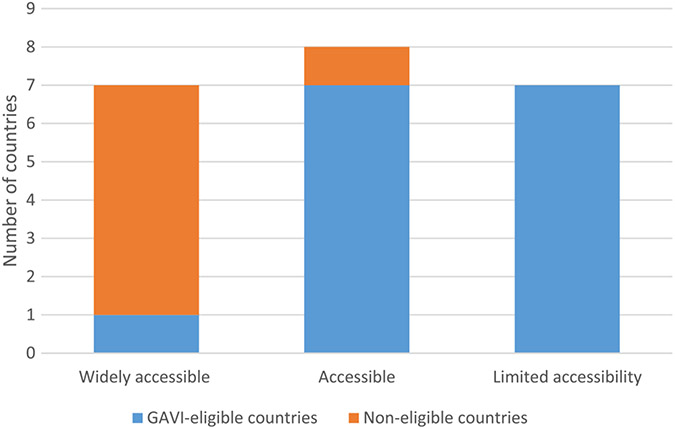
Access to rabies vaccines by Gavi-eligibility.

**Fig. 2.**
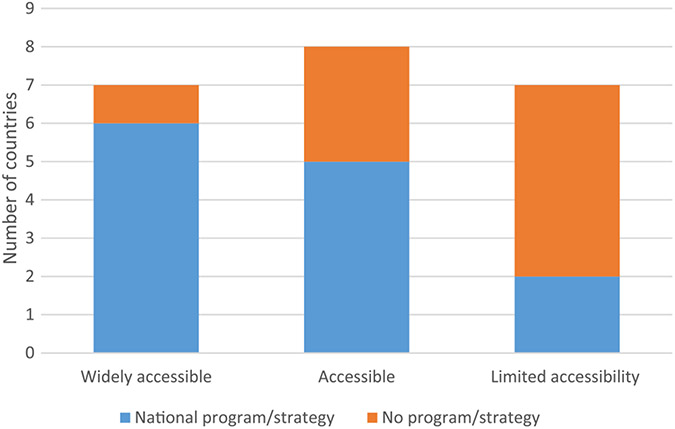
Access to rabies vaccines by presence of national rabies control program or strategy.

Of the 23 countries assessed, 22 responded to questions on vaccine access, and all 23 countries responded to questions on RIG access (Tables 1 and 2). Using the definitions described under methods, vaccine was widely accessible in 8 (36%) of 22 countries (7 in Asia and one in Africa), accessible in 7 (32%) of countries, and limited in almost a third (32%; 7/22) of countries. Of the countries with limited access to rabies vaccine, five were in Africa and two in Asia, and all were Gavi-eligible in 2017; only one country with widespread access to vaccine was Gavi-eligible (Fig. 1). RIG was found to be less accessible than vaccine, with limited access in almost two thirds (65%) of the countries assessed, 11 in Africa and 4 in Asia. Only two (9%; 2/23) countries had RIG widely accessible (Tables 1 and 2). Of the 7 countries with wide access to vaccine, 6 (86%) of the 7 had a national rabies control program or strategy. Of the 7 countries with limited access to vaccine, only 2 (29%) had a control program/strategy in place (Fig. 2). Moreover, of the 13 countries with a national program/strategy, 6 (46%; 6/13) had wide access to vaccine and only 2 (15%; 2/13) had limited access to vaccine. By contrast, among countries without a national program or strategy, only 1 (11%; 1/9) had wide vaccine access but 5 (56%; 5/9) had limited access to vaccines (Fig. 2).

### 3.4. Procurement and forecasting

All 23 countries provided information on procurement mechanisms. All countries reported procuring rabies vaccine using a separate system from that of their Expanded Program on Immunization (EPI) (Table 2). Time from procurement of the vaccine to arrival in country averaged 6 months, ranging from almost immediate delivery up to 1 year. Most countries (78%; 18/23) reported procuring PEP for the whole country at the central level, with only 5 countries reporting procurement occurring at a lower administrative level (province, region or state) or at the health facility level (Table 2). In addition, 22 (96%) of 23 countries reported using their own budget to procure PEP with only one of these countries reporting receipt of a small amount of vaccine and RIG as a one-time donation. In one country (Madagascar), a non-profit foundation has an agreement with the MoH and procures PEP for the entire country free of charge. No countries received subsidies for PEP at the central level, though some research institutions and NGOs had independent agreements with vaccine manufacturers and were able to provide PEP at a subsidized cost to patients.

We received responses from 20 countries on forecasting. Almost all countries (90%; 18/20) based rabies vaccine forecasting on previous consumption (previous month, quarter or year), with a buffer added if the budget allowed, to account for an expected increase in utilization. However, 8 (44%) of these 18 countries reported that they could not meet the forecasted need due to a lack of funds or limited production capacity, and instead procured based on budget availability. The remaining two countries, consistently procured rabies vaccine based on the available budget. Of the 7 countries with wide access to vaccine, 6 countries forecasted based on previous consumption and were consistently able to meet the forecasted needs; 1 country with wide access could not meet the forecasted needs to due to a lack of funds. Similarly, of the 8 countries that reported not being able to meet forecasted needs due to budget constraints, 1 (13%; 1/8) country had vaccine widely accessible, 4 (50%; 4/8) countries had vaccine accessible, and 3 (38%; 3/8) countries had limited vaccine accessibility. There was no data available on the proportion of patients seeking PEP who received it in the public sector and those that were unable to due to a lack of vaccine stock.

### 3.5. Vaccine type, schedule and administration route

Of the 23 countries assessed, 22 provided information on vaccine type, while 23 provided information on schedule and administration route. Only 6 (26%) of 22 countries reported exclusively using prequalified rabies vaccines in the public sector, whereas more than two thirds of countries (68%; 15/22) also used non-prequalified vaccines because of their lower cost or increased availability (Table 2). Three countries manufacture cell culture-based vaccine; all three export vaccine to other countries. One country manufactures and uses nerve tissue-based vaccine.

The most common administration route for rabies vaccination, used by 18 (78%) of 23 countries, was IM (Tables 1 and 2). Of these 18 countries, 12 (10 in Africa, 2 in Asia) used IM administration exclusively at all public health facilities providing PEP, whereas five countries, all in Asia, reported using both ID and IM administration depending on the hospital, province or region (Tables 1 and 2). One country used subcutaneous administration for the nerve-tissue based vaccine and IM administration for CCEEVs. Of the 18 countries using IM (exclusively or non-exclusively), 15 (83%; 15/18) countries reported using the Essen 5-dose schedule (Table 1). Five (22%; 5/23) countries, four in Asia and one in Africa, reported exclusively using ID administration throughout the country in the public sector. As of 2017, all countries using ID administration reported using the updated Thai Red Cross schedule (Table 1) [5].

### 3.6. Distribution and storage

We received information on vaccine storage and distribution from 21 and 22 countries respectively. At the time of the survey, two countries were in the process of transitioning away from using the same distribution system as the EPI program used for other vaccines. Half of all countries assessed (50%; 11/22) had a standardized distribution or collection system for PEP, separate from the systems used for EPI vaccines, with frequency of distribution ranging from monthly to annual (Table 2). These systems were not necessarily dedicated to rabies vaccines alone, but could be combined with other (non-EPI) pharmaceutical distribution. Some countries (27%; 6/22) had an ad hoc system of collection/distribution separate from EPI distribution systems depending on vaccine availability at the central level (due to limited funds to purchase vaccine) or need for vaccine at lower levels. In four countries (18%; 4/22), rabies vaccines were distributed regularly through a combination of the manufacturer and the MoH. In one country, manufacturers or in-country distributors were entirely responsible for distributing PEP directly to health facilities (Table 2).

Approximately half of the countries (52%; 11/21) used the same storage facilities for rabies and other EPI vaccines at some level of the health system but typically not at every level; for example, refrigerators may be shared at some health facilities, or warehouses may be shared at the central level (Table 2). The remaining countries assessed (48%; 10/21) used separate storage facilities for rabies vaccines, with no overlap between EPI vaccines and rabies vaccine, either because rabies vaccination was provided in separate facilities or because national EPI guidelines did not allow non-EPI vaccines to be stored with EPI vaccines.

### 3.7. Monitoring and reporting

Of 23 countries, 22 provided information on monitoring, reporting, and tracking defaulters (those that miss scheduled vaccinations). Almost all countries (86%; 19/22) reported having some type of monitoring system in place for tracking patients receiving PEP or for monitoring vaccine stock (Table 2). However, in at least 6 (32%; 6/19) of these countries, the monitoring and reporting tools were not nationally standardized, their use was not enforced, or use was limited to selected health facilities only. As a result, variables collected varied greatly by country, and in some cases, by health facility within a country. Variables collected included number of patients receiving PEP, number of bite patients seen in a health facility, and number of vials of vaccine used. The remaining three (14%; 3/22) countries did not have a monitoring system in place.

Almost half (41%; 9/22) of all countries assessed reported having regular weekly, monthly or quarterly reporting on rabies vaccination. Similar to monitoring variables, variables reported varied across countries and included the number of people vaccinated, the number of vials used, or the number of doses used. An additional five countries (23%; 5/22) had reporting systems that were poorly enforced, irregular and/or not mandatory in all health facilities. In 8 (36%) of 22 countries, there was no reporting on rabies vaccination (Table 2).

Of 22 countries, 11 (50%) had a system for monitoring adverse events following PEP (Table 2). However, at least 3 (27%; 3/11) of these countries reported the system being weak or limited to capturing major adverse events. The remaining countries did not have a system for monitoring adverse events following PEP. A majority of countries (86%; 19/22) had no or very limited systems for tracking patients who had not completed a full course of PEP (Table 2).

## 4. Discussion

Though all 23 countries assessed in Asia and Africa have rabies vaccines available to some extent, broad and affordable access to vaccine is limited. Key barriers to access to PEP continue to be the high cost and limited availability of the vaccine particularly at lower administrative levels outside the central level. In over half the countries assessed, patients are typically required to pay the full cost of the vaccine in the public sector (either due to national policy or budget constraints resulting in unavailability of vaccine), and in almost a third of countries, access to vaccine is geographically restricted to a few locations only. Over two-thirds of countries reported using non-prequalified vaccines. Among all countries, IM administration is the most common administration route.

The primary reason for the high cost of the vaccine to the patient is a lack of funding for rabies prevention and control at the government level. All countries with limited access represented here are Gavi-eligible and are thus low-income, with a GNI per capita below or equal to US$ 1580 on average over the past three years [11]. With over 15 non-prequalified vaccines on the market and only four pre-qualified rabies vaccines, non-prequalified vaccines are often cheaper and more widely available [13,14]. Vaccine manufacturers are encouraged to submit their vaccines for WHO prequalification, as prequalification provides an additional quality assurance that a vaccine is safe and effective. Moreover, WHO prequalification is required by some organizations (e.g., Gavi), that invest in vaccines.

RIG was not available in most countries we assessed and even countries that have RIG, report prioritizing its use because of its high cost and limited global availability. Most countries reported prioritizing RIG for wounds that are more severe, located near or on the head and neck, or wounds in younger children, which is in line with the WHO recommendations published in 2018, emphasizing the use of RIG for high risk patients where there is limited availability of RIG [4,15].

Of the countries assessed, very few, particularly in Africa, are currently exclusively using ID administration for rabies vaccine. The new WHO recommendations highlight the cost-effectiveness of ID administration, even in low-volume settings [4]. More importantly, the new recommendations recommend a shortened one-week, 3-visit ID schedule, which is time, cost and dose-sparing in comparison to the previous ID schedule consisting of 4 visits over two weeks [4]. ID regimens are also more cost-effective than IM regimens particularly in high-volume clinics [4,16,17]. There are several possible reasons for the lack of introduction of ID administration. First, many rabies vaccines (including all of the WHO prequalified vaccines) are not labelled for ID use, so some countries may be reluctant to use the vaccine off-label, despite an abundance of evidence indicating that the vaccine is equally safe and efficacious when administered intradermally among persons who are immunocompetent. Second, few other vaccines are administered intradermally so national introduction of this administration route for rabies vaccines would likely require additional training. However, recent experiences with fractional-dose inactivated polio vaccine administered intradermally have shown success in delivering vaccine on a large scale through alternative administration routes [18-20]. Thus, ID administration should be encouraged globally to increase availability and affordability of vaccines. Policy advocacy and capacity building activities should be initiated to approve ID administration in countries that continue to use IM administration. While changing vaccination schedules can be a challenge, the new ID schedule is an abbreviated version of the previously recommended schedule so while the first three visits remain the same, the shorter schedule should greatly assist in completion of the vaccination series [4]. Vaccine manufacturers should be encouraged to label their vaccines for ID use.

As of 2018, none of the countries surveyed are using the same system for both EPI and rabies vaccine distribution or collection. However, many countries use shared cold storage facilities at some level of the health system. Because most countries assessed have a regular system of distribution for rabies vaccines (either an independent system or combined with manufacturers delivering vaccine), it is possible that the same systems would be able to handle an increase in their distribution capacity for rabies vaccines, if additional vaccines were to be made available. Alternatively, sharing distribution networks with other systems such as the EPI system could also be considered as this may decrease logistical costs of rabies vaccine distribution, resulting in an overall lower cost for governments. In light of limited availability of PEP in most countries, alternative vaccine delivery strategies should be considered. Such strategies could include providing vaccine at one health facility per geographic area as opposed to attempting to target every facility in each geographical area, as this would simplify logistical challenges while still providing widespread availability.

Despite our finding that almost all countries have monitoring tools, less than half have mandatory reporting of rabies vaccine use or numbers of patients vaccinated, and even fewer have systems in place for tracking patients who fail to complete a course of PEP. Additionally, there is a lack of standardization of monitoring and reporting tools within and across countries resulting in poor data on bite burden, vaccine utilization, and numbers of people receiving and completing a course of PEP. There are currently no global guidelines available for countries planning to introduce or implement a rabies vaccination program. With the potential for additional investments in rabies vaccine, guidelines are needed which emphasize the importance of monitoring and reporting in order to improve data availability on vaccine needs and forecasting to guide procurement [3]. While countries would need to tailor guidelines to their context, global guidelines may encourage them to institute nationally standardized tools as well as mandatory reporting.

This assessment was subject to a number of limitations. First, the quality and level of detail varied across countries so we were not able to obtain key indicators for every variable in every country. However, the assessment was intended to provide an overview and not specific country-level data. Second, it is challenging to compare countries and programs that vary in income level. Finally, health facilities visited for the purposes of the assessment were not randomly sampled and may not be representative of the whole country.

A larger proportion of countries with established national rabies control programs or strategies had widespread access to rabies vaccines. There is a need for national rabies control programs to be established in all rabies-endemic countries. Rabies control programs could learn from the EPI, where monitoring and reporting are typically standardized and where defaulter tracking is an established part of many programs. National rabies control strategies or control programs may be instrumental to eliminating rabies; without government buy-in, and an increase in funding for both the human health and animal sectors, rabies prevention and control will remain a challenge. Although dog vaccination remains the mainstay of rabies prevention and control it requires substantial and long-term government investment; therefore, providing exposed persons with timely access to PEP is important in reducing the burden of disease in humans [21-24]. As key barriers to improved access continue to be the high cost of the vaccine to governments and as a result, to patients, investments in PEP will likely improve access for many bite victims.

## Figures and Tables

**Table 1:** Overview of administration route, dosage schedule, cost, and accessibility of rabies vaccine and rabies immunoglobulin (RIG) in the public and non-private sectors by country, in selected countries in Asia and Africa (N = 23), January 2017–May 2018.

Country	Route of administration^a^	Dosage schedule^b^	Vaccine accessibility^c^	Vaccine cost to patient	RIG accessibility^c^	RIG cost
Cameroon*	IM	Zagreb	Accessible	US$ 13–17/dose	Limited	–
Chad*	IM	Essen 5-dose	Limited	US$ 13/dose	Limited	–
Côte d’Ivoire*	IM	Zagreb Essen 5-dose	Accessible	US$ 13/dose	Limited	–
Ethiopia* ^† ‡^	Subcutaneous IM	Nerve-tissue based vaccine 17-dose Essen 5-dose	Limited	US$ 2–4/course US$ 13/dose	Limited	–
Ghana*	IM	Essen 5-dose	Limited	Free	Limited	–
Kenya* ^†^	IM	Essen 5-dose	Accessible	US$ 12–15/dose	Limited	US$ 70/vial
Madagascar* ^† ¶^	ID	Updated Thai Red Cross	Accessible	Free	Limited	Free
Mali*	IM	Essen 5-dose	Limited	US$ 20/dose	Limited	–
Malawi (Blantyre district only)*	IM	Essen 5-dose	Information not available	Free	Limited	–
Nigeria* ^†^	IM	Essen 5-dose	Limited	Free	Limited	–
South Africa^†^	IM	Essen 4-dose	Widely accessible	Free	Widely accessible	Free
Tanzania* ^†^	IM	IM 3-dose (0,7,28)	Accessible	US$ 13	Limited	–
Bangladesh* ^†^	ID	Updated Thai Red Cross	Widely accessible	Free	Accessible	Free-US $ 15/vial
Bhutan^†^	ID	Updated Thai Red Cross	Widely accessible	Free	Accessible	Free
India* ^†^	ID IM	Updated Thai Red Cross Essen 5-dose	Accessible	Free	Limited	Free
Nepal*	ID IM	Updated Thai Red Cross Essen 5-dose	Accessible	Free	Limited	–
Sri Lanka^†^	ID	Updated Thai Red Cross	Widely accessible	Free	Accessible	Free
Pakistan*	ID IM	Updated Thai Red Cross Essen 5-dose	Limited	Free	Limited	Free
Cambodia*	ID IM	Updated Thai Red Cross Essen 5-dose	Limited	Free – US$ 15/dose	Limited	US$ 37/patient
China^†^	IM	Zagreb Essen 5-dose	Widely accessible	US$ 50/coursed^d^	Widely accessible	US$ 25–50/vial
Mongolia	IM	Essen 5-dose	Widely accessible	Free	Limited	Free
Philippines^†^	ID	Updated Thai Red Cross	Widely accessible	Free	Accessible	US$ 28–32/vial
Vietnam^†^	ID IM	Updated Thai Red Cross Essen 5-dose	Accessible	US$ 7–13/dose	Accessible	US$ 15–27/vial

* Gavi-eligible countries; countries with a Gross National Income per capita below or equal to US$ 1580 on average over the past three years (2015–2017).

† National program or national guidelines in place.

‡ In Ethiopia, cell-culture based vaccines are available at selected public health facilities in the capital, but nerve-tissue based vaccines are more widely used. The Ethiopian national guidelines for rabies prevention and control recommend cell-culture based vaccines.

¶ In Madagascar, the Institut Pasteur de Madagascar (IPM), procures PEP for the entire country using their own budget. Through an agreement with the Ministry of Health, rabies vaccine purchased by IPM is available at public health facilities; as a result, patients are able to access PEP at public health facilities free of cost.

a ID = Intradermal; IM = Intramuscular.

b Updated Zagreb (2-site IM route of administration on day 0 and 1-site IM route of administration on days 7 and 21); Essen 5-dose (1-site IM route of administration on days 0, 3, 7, 14, 28); Nerve-tissue based vaccine 17-dose (1-site subcutaneous route of administration on days 0–13 and 10, 20, 30); Updated Thai Red Cross (2-site ID route of administration on days 0, 3, 7, 28); Essen 4-dose (1-site IM route of administration on days 0, 3, 7, and between day 14–28).

c Widely accessible = vaccine or RIG available for free or at a subsidized cost at the central level, provincial, state or regional level, and at least one health facility in every district, county or zone; Accessible = vaccine or RIG available at the central level and provincial, state, or regional level but not in every district, county or zone, or available in every district but at a cost to patients (greater than US$ 5/dose); Limited accessibility = vaccine or RIG only available at the central level (regardless of cost) or being sporadically available at lower levels because of budget constraints or stock outs.

d In China, the cost of PEP is partly reimbursable in some places.

**Table 2:** Summary description of rabies post-exposure prophylaxis accessibility, procurement, distribution, monitoring and reporting in selected countries in Asia and Africa (N = 23), January 2017–May 2018.

Characteristics	Overall		Africa*		Asia^†^	
	n	%	n	%	n	%
Total	23		12	52	11	48
*Gavi eligibility* ^a^	23		12		11	
Gavi-eligible	16	70	11	92	5	45
Gavi non-eligible	7	30	1	8	6	55
*National program*	22		11		11	
Yes	13	59	6	55	7	64
No	9	41	5	45	4	36
*Accessibility*						
Access to vaccine	22		11		11	
Widely accessible	7	32	1	9	6	55
Accessible	8	36	5	45	3	27
Limited accessibility	7	32	5	45	2	18
Access to RIG^b^	23		12		11	
Widely accessible	2	9	1	8	1	9
Accessible	5	22	–	–	5	45
Limited accessibility	16	70	11	92	5	45
*Vaccine type, schedule, and administration route*						
Vaccine	22		11		11	
WHO PQ vaccines exclusively^c^	6	27	4	36	2	18
Use non WHO PQ (exclusively or in addition to PQ vaccines)	16	73	7	64	9	82
Route of Administration^d^	23		12		11	
IM exclusively	12	52	10	83	2	18
ID exclusively	5	22	1	8	4	36
IM and ID	5	22	–	–	5	45
Subcutaneous	1	4	1	8	–	–
*Procurement and Distribution*						
Procurement	23		12		11	
Procure at central level	18	78	11	92	7	64
Procure at lower level	5	22	1	8	4	36
Distribution	22		11		11	
Standardized regular distribution	11	50	5	45	6	55
Ad hoc distribution	6	27	5	45	1	9
Combination of manufacturer and Ministry of Health distribution	4	18	1	9	3	27
Manufacturer/in-country distributor alone	1	5	–	–	1	9
Cold Chain	21		10		11	
Use entirely separate storage facilities to EPI	10	48	5	50	5	45
Share some storage facilities with EPI	11	52	5	50	6	55
*Monitoring and reporting*						
Monitoring	22		11		11	
Standardized monitoring system in place	19	86	3	27	9	82
Monitoring system but only in some places, health facility- dependent or not enforced	7	32	5	45	2	18
No monitoring system in place	3	14	3	27	–	–
Reporting	22		11		11	
Regular mandatory reporting	9	41	3	27	6	55
Irregular reporting or not mandatory	5	23	3	27	2	18
No reporting	8	36	5	45	3	27
AEFI^e^	22		11		11	
System for monitoring AEFI to rabies vaccine (even if limited)	11	50	5	45	6	55
No system for reporting AEFI following rabies vaccine	11	50	6	55	5	45
Defaulter tracking	22		12		10	
System for tracking rabies vaccine defaulters	3	14	–	–	3	30
No or limited system for tracking rabies vaccine defaulters	19	86	12	100	7	70

* Countries assessed in Africa = Cameroon, Chad, Cote d’Ivoire, Ethiopia, Ghana, Kenya, Madagascar, Mali, Malawi, Nigeria, South Africa, Tanzania.

† Countries assessed in Asia = Bangladesh, Bhutan, Cambodia, China, India, Mongolia, Nepal, Pakistan, Philippines, Sri Lanka, Vietnam.

a Gavi-eligible countries = countries with a gross national income per capita below or equal to US$ 1580 on average over the past three years (2015–2017).

b RIG = rabies immunoglobulin.

c WHO PQ vaccines = WHO prequalified vaccines.

d ID = Intradermal route of administration; IM = Intramuscular route of administration.

e AEFI = adverse events following immunization.
